# Impact of a dementia-friendly program on detection and management of patients with cognitive impairment and delirium in acute-care hospital units: a controlled clinical trial design

**DOI:** 10.1186/s12877-022-02949-0

**Published:** 2022-03-31

**Authors:** NM Weldingh, MR Mellingsæter, BW Hegna, J Saltyte Benth, G Einvik, V Juliebø, B Thommessen, M Kirkevold

**Affiliations:** 1 Division of Research and Innovation, Department of Research Support Service, Akershus University Hospital, Lørenskog, Norway; 2 Department of Geriatric Medicine, Division of Medicine, Akershus University Hospital, Lørenskog, Norway; 3 Institute of Clinical Medicine, University of Oslo, Oslo, Norway; 4 Health Services Research Unit, Akershus University Hospital, Lørenskog, Norway; 5 Department of Pulmonary Medicine, Division of Medicine, Akershus University Hospital, Lørenskog, Norway; 6 Department of Cardiology, Division of Medicine, Akershus University Hospital, Lørenskog, Norway; 7 Department of Neurology, Division of Medicine, Akershus University Hospital, Lørenskog, Norway; 8 Faculty of Health Sciences, Department of Nursing and Health Promotion, Oslo Metropolitan University, Oslo, Norway; 9 Faculty of Medicine, Department of Nursing Science, University of Oslo, Institute of Health and Society, Oslo, Norway

**Keywords:** Cognitive impairment, Delirium, Dementia-friendly hospital, Delirium screening, Delirium prevention, Delirium treatment measures, Delirium detection, Implementation

## Abstract

**Background:**

Frail older persons with cognitive impairment (CI) are at special risk of experiencing delirium during acute hospitalisation. The purpose of this study was to investigate whether a dementia-friendly hospital program contributes to improved detection and management of patients with CI and risk of delirium at an acute-care hospital in Norway. Furthermore, we aimed to explore whether the program affected the detection of delirium, pharmacological treatment, 30-day re-hospitalisation, 30-day mortality and institutionalisation afterwards.

**Methods:**

This study was part of a larger quality improvement project aiming at developing and implementing a new program for early screening and management of patients with CI. This study, evaluating the program are designed as a controlled clinical trial with a historical control group. It was conducted at two different medical wards at a large acute-care hospital in Norway from September 2018 to December 2019. A total of 423 acute hospitalised patients 75 years of age or older were included in the study. Delirium screening and cognitive tests were recorded by research staff with the 4 ‘A’s Test (4AT) and the Confusion Assessment Measure (CAM), while demographic and medical information was recorded from the electronic medical records (EMR).

**Results:**

Implementation of the dementia-friendly hospital program did not show any significant changes in the identification of patients with CI. However, the share of patients screened with 4AT within 24 h increased from 0% to 35.5% (*P* < .001). The proportion of the patients with CI identified by the clinical staff, who received measures to promote “dementia-friendly” care and reduce the risk for delirium increased by 32.2% (*P* < *.*001), compared to the control group. Furthermore, the number of patients with CI who were prescribed antipsychotic, hypnotic or sedative medications was reduced by 24.5% (*P* < *.*001). There were no differences in delirium detection, 30-day readmission or 30-day mortality.

**Conclusions:**

A model for early screening and multifactorial non-pharmacological interventions for patients with CI and delirium may improve management of this patient group, and reduce prescriptions of antipsychotic, hypnotic and sedative medications. The implementation in clinical practice of early screening using quality improvement methodology deserves attention.

**Trial registration:**

The protocol of this study was retrospectively registered in the ClinicalTrials.gov Protocol Registration and Results System with the registration number: NCT04737733 and date of registration: 03/02/2021.

## Background

The number of people over 60 years is increasing, estimated to double between 2015 and 2050 [1]. Comorbidity and impaired physical and cognitive function is more common in older age, and the patients require hospitalisations more frequently. One third of older patients presenting in the hospital emergency department have some type of cognitive impairment (CI) [2, 3]. CI is a broad term encompassing varying degrees of severity from mild cognitive impairment to dementia. Patients with CI are in high risk of developing delirium during hospital stays [4–6] and it is shown that delirium may accelerate the development of CI [7–9]. Delirium is an acute and reversible form of CI, characterised of a sudden and drastic change in the ability to focus attention and is caused by an acute medical condition [10]. The prevalence of delirium in general medical wards is estimated to be 18–35% [5]. Delirium during acute hospital stays is associated with falls, increased length of stay, admission to long-term care and increased mortality [5, 11, 12]. However, despite the frequency of the conditions and the evidence of delirium as preventable [13], screening of CI and delirium is rarely implemented as routine practice in hospital wards and in about 75% of the cases, delirium is not detected [13]. The coexistence of delirium and dementia is particularly common in hospital settings and may be difficult to distinguish [14, 15]. To differentiate between the two, knowledge of baseline status and identification of an acute change is necessary [14]. Since the risk of delirium increases with the number of risk factors present, a multi-component approach targeting the patient’s risk factors is the most effective strategy for reducing the risk of delirium [16]. Effective preventive approaches are implemented in the National Institute for Health and Care Excellence Guidelines (NICE) [17], the Hospital Elder Life Program (HELP) [18] and the Acute Care for Elders (ACE) strategy [19]. However, such effective interventions and preventive strategies have often included additional staff and volunteers [20–23] or specialized geriatric wards or consultations [11, 24]. Due to differences in the organization of health care, and the tendency to increasingly shorter lengths of stay, models may not be directly transferable to current hospital wards. Programs that are adapted and tested in routine practice of current health service organisations are therefore warranted. Results show that patients with delirium superimposed on dementia had worse long-term outcomes, including longer length of hospitalization, worse cognitive and functional outcomes, and a higher risk of institutionalization and mortality than patients with dementia [15]. This highlights the need for early identification of patients with a CI and delirium to initiate preventive and treatment strategies. User organizations and health governments have advocated a call for more “dementia-friendly” hospital services, adjusted to the needs of patients with some form of CI [25]. Therefore, a “dementia-friendly” hospital program emphasising improved care to patients with CI was designed to improve the detection, routine care and management of patients with CI and the risk of delirium in acute medical wards. In this study, the focus was not limited to patients diagnosed with dementia; we included a wider group of older individuals with some form of CI identified during the hospitalisation and/or vulnerability to delirium. This vulnerable patient group is frequently encountered in acute care settings. Although naming the program dementia-friendly, we assumed that the program would address the needs of this wider group at high risk of developing delirium during the hospital stay.

The program was developed and implemented as part of a larger quality improvement project composed of a multidisciplinary team of nurses and physicians who worked according to the principles of the quality improvement model [26] including Deming`s Plan-Do-Study-Act (PDSA) method [27]. This has been referred to as a necessary strategy when implementing and evaluating the effectiveness of new models in practice [28–30].

There was no routine screening of CI, nor systematic follow-up of patients with a risk of delirium in daily practice when the project was initiated. Therefore, quality improvement was needed to ensure adequate care of these patients. The larger project also included interviews with older hospitalized patients with an indication of CI, and their informal carers, about their experiences of an acute hospital stay (results to be published elsewhere). This knowledge contributed to developing the dementia-friendly hospital program in accordance with the older people' and relatives' reported experiences and needs.

This multi-component intervention program included implementation of an educational program for healthcare professionals, systematic screening of CI, and highlighted measures to prevent and treat delirium. The program was designed to enable implementation without additional resources and comprehensive changes of routine care, as the wards were busy and comprehensive education and practice change were not realistic. The program was also designed to raise staff awareness of CI and delirium. To evaluate this program, we carried out a comprehensive data collection before implementing the new program, which represents the historical control group in this study. Due to the quality improvement design, a randomized controlled study was not feasible.

The primary aim of this study was to explore in what degree the “dementia-friendly” program improved the detection rate of CI by clinical staff during acute hospitalisation and the initiation of relevant preventive and treatment measures. Secondary aims included impact of the program on rate of patients screened for CI by the clinical staff within the first 24 h of admission, detection of delirium, initiation of antipsychotic medications, hypnotics or sedatives, length of hospital stay, admission to a higher level of care in the primary health service after discharge, 30-day re-hospitalisation and 30-day mortality.

## Methods

### Study design

**Fig. 1:**
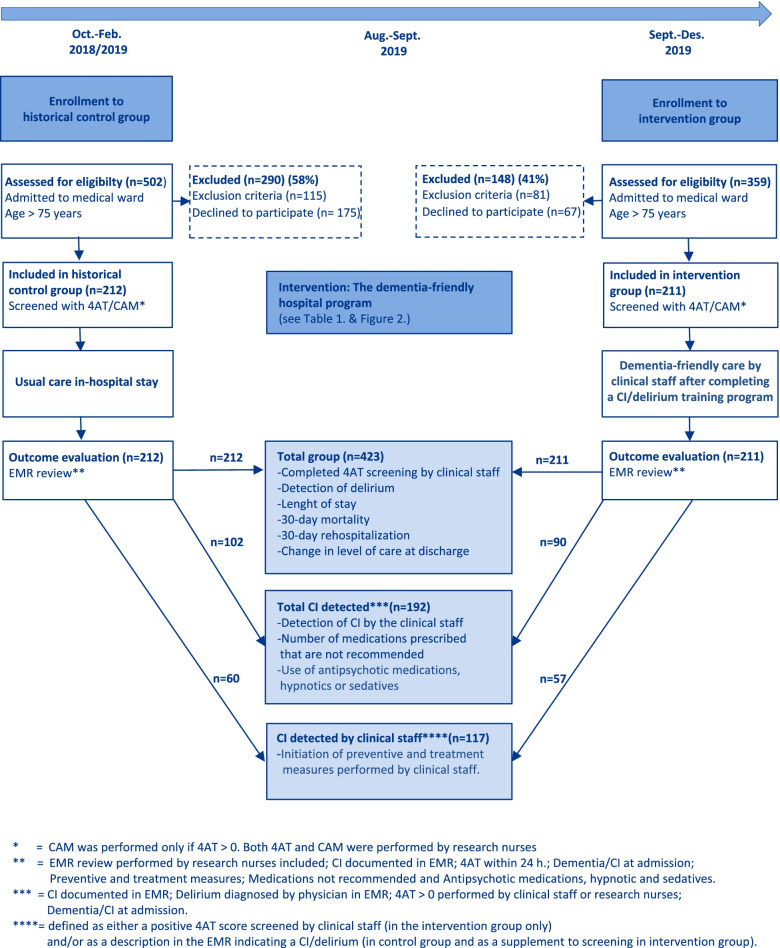
Study flow chart: Recruitment and assessment in the dementia-friendly hospital program study

This study had a controlled clinical trial design with a historical control group and was carried out at two medical wards at Akershus University Hospital, a large acute-care hospital in Norway, with a catchment area of 600.000 inhabitants. A pulmonary (27 beds) and a cardiac bed ward (28 beds) participated in this project. The control group received usual care, whereas the intervention group received usual care plus the dementia-friendly program. A study flow chart (Fig. 1) summarises the design of the study.

### Study sample

Patients 75 years of age or older, admitted to one of the participating wards for acute medical illness between October 2018 and February 2019 (control group) and between September 2019 and December 2019 (intervention group), were eligible. Exclusion criteria included critical illness, inability to communicate (whether from aphasia, severe hearing loss, or inability to speak Norwegian) or isolated because of severe infections. Patients were only included once, implying that readmitted patients enrolled during a previous hospital stay were excluded.

### Intervention: the dementia-friendly hospital program

The dementia-friendly hospital program was based on the National Institute for Health and Care Excellence (NICE) delirium guidelines [17] and the HELP program [31] and was reviewed for relevance by an advisory board for dementia at the National Association for Public Health [32] and the Oslo Delirium Research Group [33]. The program comprised three parts, which are illustrated in Table 1:

1. **Educational program for health practitioners**
  An educational program was developed to increase the staff’s knowledge and awareness of patients with CI and/or delirium and to support the implementation of the program.
2. **Screening of CI and delirium**
  For early identification of CI and delirium, the program included screening by the clinical nurses within 24 h after admission to the medical wards using the 4 ‘A’s Test (4AT) [34]. The clinical nurses were encouraged to screen beyond 24 h if they did not manage within 24 h.
3. **Interventions to prevent and manage delirium**

**Table 1:** The dementia-friendly hospital program

**Educational program for health practitioners**	
Digital educational electronic course	How patients with CI may experience hospital admission Detection of patients with CI and delirium Delirium-prevention treatment strategy and follow-up of CI
‘Nurse-champions’	Three local ‘nurse-champions’, on each ward
Morning lecture	30-min lecture for the ward physicians by a geriatric specialist
Pocket-sized handouts	Visualizing the 4AT screening tool, the multi-component preventive interventions and delirium management suggestions
**Screening of cognitive impairment and delirium**	
4AT screening within 24 h after admission to the ward (If 4AT screening is not managed within 24 h, screening should be performed as soon as possible)	
**Interventions to prevent delirium**	
Orientation	Orienting communications
	Ensure patient has eyeglasses and hearing aids, if needed
Nutrition and hydration	Early recognition of dehydration and risk of malnutrition
	Encouragement of oral intake of fluids and encouragement during meals
	Early correction of hypovolemia and electrolyte imbalance
Elimination	Prevent obstipation (e.g. encourage regular toilet routines)
	Early recognition of urinary retention (e.g. bladder scanning)
Mobilisation	Encourage daily mobilisation adapted to previous functional level
	Avoid restraints and immobilising equipment if possible (e.g. Foley catheters)
Sleep hygiene	Noise and light reduction at night
	Reschedule procedures to allow at least five hours of uninterrupted sleep at night
	Cognitive stimulation to reduce sleeping during the day
Pain management	Assess nonverbal signs of pain
	Optimize pain management, preferably with nonopioid medications
Medications review	Review the patient’s medication list to reduce polypharmacy and to avoid any medications associated with precipitating delirium (e.g. benzodiazepines, antihistamines, high dose of opioids)
Family involvement	Facilitate presence of relatives when giving important information to the patient
	Facilitate presence of relatives outside visits
**Management of delirium**	
Identify and treat underlying causes	Search for infections, metabolic abnormalities and acute pain and treat as appropriate Assess polypharmacy and side effects of medications
Reduce contributing factors and optimise orienting factors	Maintain preventive measures to optimise orientation and reduce contributing factors for delirium (e.g. stabilise vital abnormalities)
	Increase continuity of care by reducing number of nurses caring for patient
	Place patient in single room if possible
	Early assessment of need for 24-h nursing; facilitate the presence of relatives
Prevent complications	Prevent aspiration pneumonia, pressure sores, deep venous thrombosis and falls
Pharmacological strategies	Procedure with preferred use of type and dosage of antipsychotics to manage severe agitation
	Manage sleep–wake cycle
Family involvement	Offer conversation with patients and relatives to inform them about delirium and follow-up after the delirium
Cognitive assessment	Referral to assessment of cognitive function after discharge

**Fig. 2:**
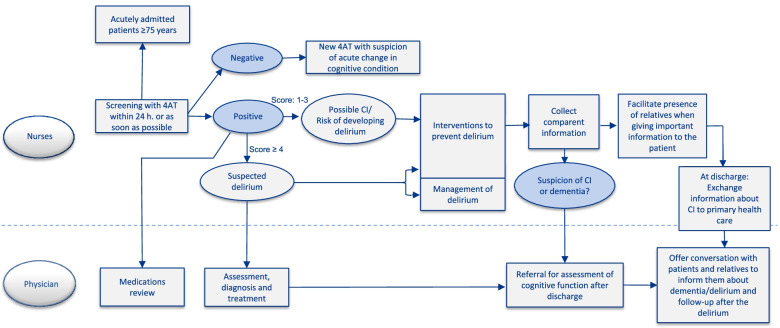
The dementia-friendly hospital program

A 4AT score > 0 was defined as potential CI and risk of delirium, implying that preventive measures should be implemented. For those with a 4AT score ≥ 4 and suspicion of delirium, the program promoted an additional delirium management plan. The main elements of the interventions program are summarised in Table 1 and in Fig. 2.

### Data collection

The data collection was performed by three research nurses trained for the study.

#### Demographic data

Demographic data, such as age, gender, place of residence (home, adapted housing, institution), and family/relative network were obtained upon admission to the study.

#### Medical data

Medical data, such as cause of admission, comorbidities, medications and medical treatment, were obtained both at admission and from their electronic medical records (EMR) after discharge. Vital signs and severity of medical condition were obtained from the National Early Warning Score2 (NEWS2) [35] upon admission to the emergency unit. The NEWS2 score uses well-established vital parameters on respiratory rate, oxygen saturation, temperature, systolic blood pressure, pulse rate, and level of consciousness or new confusion to identify patients at risk of a worsening condition. NEWS2 scores range from 0–24, where a higher score indicates higher clinical risk [36]. A score > 7 indicates a high severity and requires continuous monitoring of vital parameters.

#### Primary outcome

The primary outcome, detection rate of CI, was compared between the control group and the intervention group and were defined as the percentage of CI detected by the clinical staff during usual care of the total CI in the sample detected by the research staff. We did not distinguish between acute CI/delirium, chronic CI/dementia or delirium superimposed on dementia. CI in the total sample was defined as all patients with either a diagnosis of CI at admission, CI recorded in the EMR during the hospital stay or a positive screening by the research staff. CI detected by the clinical staff in the intervention group was defined as either a 4AT score > 0 detected through screening and/or as a description in the EMR indicating that the patient had some form of CI. In the control group, 4AT screening was not yet implemented. Thus CI detected by the staff had to be defined as a description in the EMR indicating that the patient had some form of CI/delirium.

The screening tools used by research nurses to identify CI were: the 4 ‘A’s Test (4AT) [34]. The four ‘A’s in 4AT stand for Arousal, Attention, Abbreviated Mental Test – 4, and Acute change [34]. 4AT is a brief orientation measure which include cognitive screening sensitive to cognitive impairment, in addition to items on altered level of alertness and change in mental status, which are strong indicators of delirium [34]. The 4AT instrument is validated [37–39] and consists of four variables: alertness, shortened mental assessment, attention, and acute change or fluctuating course. A score of four or more indicates delirium, while a score of 1–3 indicates CI [37]. A positive 4AT score (> 3) has shown a sensitivity and specificity of detecting delirium of 78% and 95%, respectively [34]. The 4AT has also shown a high specificity but low to moderate sensitivity with general cognitive impairment [34]. The tool also allows assessment of drowsy patients and delirium superimposed on dementia [40, 41].

To identify CI in the total sample in each group (control and intervention), 4AT was performed in all participants by trained research nurses during the first three days after patients were admitted to the ward. The clinical staff were blinded for the assessments made by the research team.

Identification of detected CI from the EMR was based on systematic review of all patient records, looking for entries that documented that the patient was assessed to have a CI. Examples of entries categorized as “CI detected by the clinical staff” in the EMR included: “The patient is not oriented for time, place or own person”; “the patient seems / is confused”; “The patient sees / hears things that are not real”; “Conversation with relatives suggests a gradual development of CI or an acute deterioration of the patient's normal cognitive function”.

The other predefined primary outcome was the type and extent of preventive and/or treatment measures against delirium the patients with detected CI received during their stay. For all participants with CI detected by the clinical staff, a thorough review of the EMR was conducted after discharge to record if, and in case of yes, which type of preventive or treatment measures were provided (according to the categorical measures in the dementia-friendly hospital program, Table 1).

#### Secondary outcomes

The percentage of patients screened with the 4AT by the clinical nurses within 24 h after admission, reflected adherence of the intervention (implementation of the dementia-friendly program) and was based on information from the EMR.

The outcome “detection of delirium” was defined as the difference in proportion of patients diagnosed with delirium by physicians, between the control group and intervention group. Even though the 4AT score can help rule out delirium and has been shown to be effective in detecting delirium [39], 4AT is not a diagnostic tool and a more thorough follow up assessment of patients with a score indicating delirium is often recommended [38]. In this project, the 4AT screening by the clinical nurses worked as a decision support for the nurses in that they communicated the symptoms and a 4AT score indicating delirium to the physician, who decided whether further diagnosis should be initiated. The research staff used the Confusion Assessment Method (CAM) [42] to verify delirium when a 4AT score indicated possible delirium (4AT score of ≥ 4). Positive CAM registered by the research staff was used to control for delirium not diagnosed by the physician. CAM is the most used, validated diagnostic tool for diagnosing delirium [42, 43]. The CAM has shown some different results in specificity and sensitivity. Mariz et al. [43] showed a sensitivity of 94% to 100% and a specificity of 90% to 95% for detecting delirium. A more recent study by MacLullich et al. [34] showed a specificity of 100% (95% CI 98% to 100%) and a sensitivity of 40% of detecting delirium. The tool consists of two parts: part one screens for overall CI and part two includes assessment of: (1) acute onset and fluctuating course; (2) inattention; (3) disorganized thinking; and (4) altered consciousness level. If all the features of (1) and (2), as well as either (3) or (4), are met, the diagnosis of delirium is likely (positive CAM) [43].

Other secondary outcomes included: The number of medications not recommended for persons at risk of delirium, that were prescribed during admission and after discharge (Table 2); new prescriptions of antipsychotic medications, sedatives or hypnotics during the hospital stay, length of stay (number of days from admission to discharge), different needs of care at discharge (departure to home, home with home nursing, short-term stay/rehabilitation stay or institutional care/nursing home), 30-day readmissions from date of discharge and 30-day mortality. All information about secondary outcomes were collected through EMR review by the research staff after discharge.

**Table 2:** Medications recommended avoiding for people at risk of delirium [39–42]

Medications	Recommendation
Tricyclic antidepressants	Should be avoided
Antipsychotic medications	High-dose antipsychotic medications should be avoided; if necessary, haloperidol, risperidone or quetiapine can be used
Histamine antagonists	Hydroxyzine and alimemazine are not recommended
Corticosteroids	Use caution with high-dose corticosteroids
Anticholinergic and beta-3 adrenergic agonist	Oksybutynin/tolterodine/solifenacin/ Darifenacin/fesoterodine/Mirabegron are not recommended
Benzodiazepines	Should be avoided, but do not quit abruptly after prolonged use
Opioid analgesics	Not recommended, but can/must sometimes be used
Metoclopramide	Not recommended but can/must sometimes be used
Clomethiazole	Could be used to induce sleep at night
Others	Caution with Digoxin and Lithium; monitor S-concentration

#### Data management

Study data were managed using the REDCap (Research Electronic Data Capture) tool hosted at the acute-care hospital [44, 45]. REDCap is a secure, web-based software platform designed to support data capture for research studies, providing 1) an intuitive interface for validated data capture; 2) audit trails for tracking data manipulation and export procedures; 3) automated export procedures for seamless data downloads to common statistical packages; and 4) procedures for data integration and interoperability with external sources.

### Statistical analysis

The study sample size was decided based on the outcome ‘proportion of patients with CI receiving delirium preventive and treatment measures’. In lack of empirical data, the protocol pre-specified that the final sample size would be based on the preliminary findings during the data collection in the historical control group, which at that time were low and equal to 10%. With the dementia-friendly hospital program, we expected that the proportion of patients receiving preventive treatment will increase to at least 50%. To show that a difference between the proportions of patients receiving preventive treatment before and after intervention is significant, according to χ^2^-test at the level of 5% and with the power of 80%, it is sufficient with approximately 25 patients in each group. As the data in the control group were already collected, it was decided to include equally many patients in the intervention group, enabling sufficiently large datasets to analyse the secondary outcomes.

The demographic and clinical patient characteristics were presented as means and standard deviations (SD) for continuous variables and as frequencies and percentages for categorical variables. Categorical variables were compared between the intervention and control groups by χ^2^-test, while the independent sample t-test was used for comparison of continuous variables. All tests were two-sided, and results with *P*-values below 0.05 were considered statistically significant. All statistical analyses were performed using SPSS v.26.

### Ethical considerations

Ethical approval for the study was obtained from the local officer for data protection and the Regional Committee for Medical and Health Research Ethics (2018/666). All patients were asked to give written consent to the use of data collected in this project. A close relative was asked to provide proxy consent if the patient was considered not able to consent.

## Results

### Sample

There were 211 and 212 patients in the intervention group and control group, respectively. The characteristics of the patients in each study group at the time of admission are shown in Table 3. The mean age and number of patients with dementia or CI established prior to admission were similar in the two groups. There were more women and more patients diagnosed with pulmonary diseases (66.5 vs. 43.1%), infections (41.0 vs. 10.9%), heart failure (90.6 vs 83.9%), and mental illness (7.5 vs. 2.8%) in the control group compared to the intervention group.

**Table 3:** Demographic and clinical characteristics of patients, *N* = 423

Characteristics	Control *N* = 212	Intervention *N* = 211	*p*-value^1^
Age			
Mean (SD)	82.6 (5.1)	82.4 (5.2)	0.656^2^
Gender			
Female, n (%)	114 (53.8)	91 (43.1)	0.028
Marital status			
N	208	211	
Married/cohabitant, n (%)	116 (55.8)	97 (46.0)	0.132
Single, n (%)	11 (5.3)	12 (5.7)	
Widow/Widower, n (%)	77 (37.0)	92 (43.6)	
Separated/divorced, n (%)	4 (1.9)	10 (4.7)	
Family network			
Yes, n (%)	209 (98.6)	209 (99.1)	1.000
Admission ward			
Cardiac, n (%)	114 (53.8)	122 (57.8)	0.402
Pulmonary, n (%)	98 (46.2)	89 (42.2)	
Transferred between wards during the hospital stay			
Yes, n (%)	59 (27.8)	57 (27.0)	0.851
Level of health care at admission			
No health care services, n (%)	39 (18.4)	54 (25.6)	0.061
Other health care services, n (%)	20 (9.4)	38 (18.0)	0.011
Weekly home nursing care, n (%)	14 (6.6)	14 (6.6)	1.000
Daily home nursing care, n (%)	47 (22.2)	61 (28.9)	0.119
Long-term institutional care, n (%)	4 (1.9)	3 (1.4)	0.703
Rehabilitation/short term stay, n (%)	11 (5.2)	5 (2.4)	0.126
Comorbidities at admission			
No diagnosis before admission	0	2 (0.9)	-
Heart failure, n (%)	192 (90.6)	177 (83.9)	0.030
Pulmonary disease, n (%)	141 (66.5)	91 (43.1)	< 0.001
Cancer, n (%)	28 (13.2)	37 (17.5)	0.225
Endocrinologic disease, n (%)	59 (27.8)	59 (28.0)	1.000
Skeletal fracture, n (%)	7 (3.3)	4 (1.9)	0.359
Infection, n (%)	87 (41.0)	23 (10.9)	< 0.001
Dementia/cognitive impairment, n (%)	12 (5.7)	13 (6.2)	0.837
Mental illness, n (%)	16 (7.5)	6 (2.8)	0.029
Other diagnosis, n (%)	161 (75.9)	116 (55.0)	< 0.001
Symptoms of acute functional impairment 2 weeks before admission, n (%)	206 (97.2)	209 (99.1)	0.312
NEWS at admission			
Mean (SD)	3.9 (2.9)	3.3 (2.7)	0.021²
Cognitive function (4AT) at admission, cat			
No cognitive impairment (4AT = 0), n (%)	132 (62.3)	150 (71.6)	0.008
Suspicion of cognitive impairment (4AT = 1–3), n (%)	61 (28.8)	55 (26.5)	
Cognitive impairment or delirium (4AT ≥ 4), n (%)	19 (9.0)	5 (2.4)	

^1^*P*-value for χ^2^-test unless otherwise specified, ^2^*P*-value for Independent samples t-test

### Detection and screening of CI

The percentage of patients detected with CI by the clinical staff of the total sample of patients detected with CI was similar in the intervention and control groups. The clinical staff detected 57 patients with CI of 90 patients detected by research staff (63.3%) in the intervention group and 60 of 102 patients (58.8%) in the control group (*P* = *0.5*23*)*. After implementing 4AT screening as part of the dementia-friendly hospital program in the intervention group, there was an overall improvement in 4AT screenings from 0 to 46.4% (*P* < 0.001). 35.5% of the patients were screened within 24 h after admission.

### Management of patients with cognitive impairment and delirium

Among those patients with CI detected by the clinical staff, significantly more patients in the intervention group than in the control group had documented interventions to prevent and/or manage delirium, according to the implemented dementia-friendly hospital program, 77% and 45% respectively (data shown in Table 4). Furthermore, there was a higher number of measures documented in the intervention group (132 measures) than in the control group (52 measures) (mean measures per patient were 2.3 (SD 2.1) and 0.9 (SD 1.4), respectively, (*P* < *0.0*01)). Table 4 shows the distribution of the number of patients identified with CI by the clinical staff, receiving the different categories of measures. Several of the categories increased significantly following the implementation of the dementia-friendly hospital program. The category ‘Family involvement’ had the highest increase, as 3.3% in the control group and 42.1% in the intervention group had documented measures in this category (*P* < *0.0*01).

**Table 4:** Documented preventive and treatment measures for patients identified with CI by the clinical staff

Characteristics	Control *N* = 60	Intervention *N* = 57	*P*-value^1^
Documented measures in the EMR			
n (%)	27 (45.0)	44 (77.2)	< 0.001
Orientation	5 (8.3)	14 (24.6)	0.016
Nutrition and hydration	2 (3.3)	14 (24.6)	0.001
Elimination	3 (5.0)	10 (17.5)	0.030
Stabilized vital abnormalities	1 (1.7)	1 (1.8)	0.971
Mobilization	2 (3.3)	11 (19.3)	0.005
Sleep hygiene	2 (3.3)	12 (21.1)	0.003
Pain management	0	4 (7.0)	0.038
Family involvement	2 (3.3)	24 (42.1)	< 0.001
Medications review	3 (5.0)	1 (1.8)	0.326
Primary nursing	2 (3.3)	3 (5.3)	0.608
Single room	6 (10.0)	2 (3.5)	0.156
Referral to cognitive assessment	12 (20.0)	5 (8.8)	0.078
Other follow-up related to suspicion of CI	12 (20.3)	31 (54.4)	< 0.001
Number of measures, mean (SD)	0.9 (1.4)	2.3 (2.1)	< 0.001^2^

^1^Patients identified with CI by the clinical staff was defined as either a positive 4AT score screened by clinical staff (in the intervention group only) and/or as a description in the EMR indicating a CI/delirium (in control group and as a supplement to screening in intervention group), ^2^*P*-value for χ^2^-test unless otherwise specified, ^3^*P*-values for independent samples t-test

### Use of antipsychotic, hypnotic or sedative medications

The proportion of all patients with CI who received antipsychotic, hypnotic or sedative medications during their hospital stay was significantly reduced from 41.2% in the control group to 16.7% in the intervention group (*P* < 0.001, Table 5).

**Table 5:** Patients with CI given doses of antipsychotic, hypnotic or sedative medications during the hospital stay

Characteristics	Control *N* = 102	Intervention *N* = 90	*P*-value
Patients with CI given doses of antipsychotic, hypnotic or sedative medications			
n (%)	42 (41.2)	15 (16.7)	< 0.001^1^
1 medication, n (%)	20 (19.6)	6 (6.7)	
2 medications, n (%)	6 (5.9)	4 (4.4)	0.004^1^
3 medications, n (%)	11 (10.8)	2 (2.2)	
4 medications or more, n (%)	5 (4.9)	3 (3.3)	
Mean (SD)	1.1 (2.3)	0.4 (1.0)	0.007^2^

^1^*P*-value for χ^2^-test, ^2^*P*-value for independent samples t-test

Among all patients with CI, there was a tendency to reduced use of benzodiazepines and opioids at discharge, controlled for use of corresponding medication at admission. Benzodiazepines were the most frequently used of the “not recommended medications” (Table 2), and were used by 41 of the 102 patients with CI in the control group (40.2%) and 30 of 90 patients with CI in the intervention group (33.3%) at discharge (*P* < *0.3*26). There were no significant differences between the intervention group and the control group in the use of other antipsychotic medications, hypnotics or sedatives.

### Departure to rehabilitation or nursing home

There was no difference in institutionalisation after discharge between the control group and the intervention group. Among patients receiving home nursing care at the time of admission, there was a 16.9% increase in departure to rehabilitation or other types of short-term stay in a nursing home in the intervention group compared to the control group.

### Length of hospital stay, delirium, readmissions and mortality

Length of hospital stay decreased by 0.8 days in the intervention group (5.4 days) compared to the control group (6.2 days). However, this change was not statistically significant (*P* < *0.1*52). The number of patients diagnosed with delirium (entry in EMR by physician) (10 vs.7 in the control group and intervention group, respectively) and number of patients with positive CAM screening performed by research nurses (11 vs.3 in the control group and intervention group, respectively) were too low for statistical analyses. There were no differences regarding 30-day readmission (49 of 212 (23.1%) in control group vs. 44 of 211 (20.9%) in intervention group) (*P* < *0.5*75) or 30-day mortality (13 of 212 (6.1%) in control group vs. 20 of 211 (9.5%) in intervention group) (*P* < *0.1*99).

## Discussion

### Detection and screening of cognitive impairment

This study assessed the implementation of a quality improvement project concerning detection and management of CI and risk of delirium in cardiac and pulmonary bed wards. Our primary aim was to improve the detection of CI by implementing a dementia-friendly hospital program, as patients with CI is in high risk of developing delirium. Implementation of the program improved the overall detection of patients with CI only slightly, and the change was not statistically significant. To interpret these results, it is important to note that the control group seemed to have somewhat poorer health than the intervention group, with higher number of suspected CI documented at admission, which may have led to more CI detected in the control group. A secondary outcome was to implement early systematic screening with 4AT. The 4AT instrument was not known or used in the wards at baseline. After implementing the program, the overall 4AT screening by clinical staff during the admission was 46.4%. 35.5% of the patients were screened within 24 h after admission. One reason why the clinical staff only screened close to half of the patients in the intervention group could be due to problems reaching out to all the staff with information about the program. Vacancies were covered by new staff who had not been trained to conduct the screening. In addition, during the implementation period, high workloads might have reduced the time available to complete the screening because the staff had to prioritize care of acutely ill patients. Thus, they might have omitted screening patients who were assumed to have normal cognitive functions.

Previous studies aiming to improve the use of 4AT screening have shown a 21–64% improvement of screenings [46–49]. However, Bearn et al. [48] was the only study with a baseline screening of 0%, as in our study, implying that the 4AT instrument was already known and in use by the healthcare staff in the other studies. We assume that implementation of a new screening instrument requires more resources and commitment. Despite Bearn et al. [48] showing an overall improvement in 4AT screenings from 0 to 64%, they only included screenings of newly confused patients, and the sample size was small. In our study, we intended to screen every patient 75 years and older, which means that many of them did not show signs of CI/delirium. Another explanation of the differences from our study may be the 13 weeks longer study period in the study by Bearn et al. [48]. We experienced a prolonged and gradual incorporation of screenings into the daily routines due to time constraints and staff turnover, creating challenges in reaching out with training and information to all staff members. We assume that the share of patients screened would have been higher with a longer implementation and study period.

### Management of patients with cognitive impairment and delirium

Implementing the dementia-friendly program in this study improved management of patients detected with CI by 32%, compared to the control group (Table 4). Patients detected with CI received more delirium preventive measures and communication with the informal careers’ improved during hospitalisation after implementation of the program. In the control group, the EMR indicated that more focus was put on referrals for further assessment of cognitive functions during the hospitalisations or in the community care than on preventing delirium during the hospitalisation. This may indicate that the dementia-friendly hospital program increased nurses’ awareness of CI as a risk factor for developing delirium and the importance of differentiating delirium from dementia. Early screenings may also have led to earlier measures and follow-up during the hospitalisations. The results from this study do not tell to what extent delirium preventive measures may prevent incident delirium or reduce the prevalence of delirium. Furthermore, there will be cases where there is a need to give antipsychotics even though preventive measures have been used. However, the results support that the dementia-friendly program increased the use of non-pharmacological interventions, which is recommended in the literature [17–20]. Results from this study contribute to the literature, showing that educational programs can improve the management of patients with CI and delirium [49–51]. Qualitative interviews with relatives of patients with CI emphasise the importance of building good partnerships with family careers’ [52]. The dementia-friendly program, including the e-learning course, may have improved the health professionals’ understanding of the importance of partnerships between the health care services and families. However, further studies are needed to explore this assumption.

### Use of antipsychotic, hypnotic and sedative medications

Antipsychotic, hypnotic and sedative medications have potentially deliriogenic effects [53, 54]. The dementia-friendly program tested in this study showed a 24.5% reduction in prescription of antipsychotic, sedative and hypnotic medications for patients with CI. These results may indicate that the health personnel had more knowledge and, thus, were more careful in using such medications. The program emphasises the importance of using non-pharmacological interventions rather than drugs. These results are in line with results from previous trials exploring the effects of non-pharmacological interventions targeted at delirium risk factors [21, 55]. Chong, Chan [55] explored the effects of a program based on core interventions from the HELP program and detected a lower use of antipsychotic medications in the patients at the unit where these interventions were implemented.

### Departure to rehabilitation or nursing home

Several trials have studied the effects of HELP-related models on institutionalisation, but with ambiguous results [20]. Our study shows no difference in overall admissions to long-term institutions at nursing homes. However, there was an insignificant increase in departures to rehabilitation or other types of short-term stays in nursing homes for the patient group receiving home care. Institutionalisation is often described as a negative result in the literature. On the other hand, discharge to rehabilitation or short-term stays may indicate better patient care because the patient’s need for care and/or rehabilitation has been identified. The results may also be seen in connection with another result in this study, as identification of CI and improved communication with families may lead to increased knowledge about the needs of this patient group. The results must be carefully interpreted considering the organisation of the Norwegian health system, where nursing homes often include both long-term and short-term care. Acute hospitals often lack capacity and must discharge patients as soon as their acute medical needs are resolved. Thus, the patients often receive short-term stays at nursing homes in anticipation of being able to return home to the same level of care as before their hospital admissions.

### Length of hospital stay

The effectiveness of multi-component non-pharmacological interventions in reducing the length of hospital stays has been studied in several trials [20, 56]. However, the results are ambiguous. In this study, the length of hospital stay showed an insignificant reduction of 0.8 days in the intervention group compared to the control group. This result is in line with the meta-analysis by Hshieh, Yang [20] on the effectiveness of the HELP program, showing an insignificant mean reduction of 0.24 days in the intervention group. The difference was not statistically significant; however, the mean length of stay for patients ≥ 75 years in the two participating medical wards was only 4.1 days after the intervention, and thus a reduction of 0.8 days may be of clinical importance.

### Delirium, readmissions and mortality

The number of patients with delirium diagnosed by physician were too low for statistical analyses. A possible explanation might be that the dementia-friendly program was not sufficiently integrated among the physicians responsible for reporting a delirium diagnosis in the EMR. However, the number of patients with positive CAM registered by the research nurses were also too low for analysis, either suggesting a low rate of delirium in the sample or that delirium was not present at the time of screening by CAM. As delirium is a variable state, it is difficult to detect unless focused attention is directed to the condition around the clock. This was not possible for the research nurses in this study for capacity reasons. Thus differences in delirium detected by CAM and delirium diagnosed by physician could not be examined. No difference in the number of patients diagnosed with 30-day readmissions to the hospital or 30-day mortality was found. The results differ from a meta-analysis by Hshieh, Yang [20], which reports significant reductions in delirium incidences after implementation of HELP-based interventions. However, other studies suggests that effects of delirium-preventive interventions on outcomes, like readmissions and mortality rates, are ambiguous [57].

### Strengths and limitations

Strengths of the study include that the clinical personnel were blinded for the assessments made by the study team, and the assessments were made during real-world clinical practice both before and after the quality improvement project. Our study has some limitations. First, this study did not have a RCT design which is considered the gold standard when testing interventions. Due to the quality improvement design, we chose a controlled clinical trial design with a historical control group to explore the impact on the dementia friendly program. Second, because screening with 4AT was not part of usual care prior to the intervention, CI detected by the clinical staff in the control group is based only on documentation in the EMR reporting of some form of cognitive impairment. Delirium diagnosis was also based on reviews of the physician documentation of delirium in the EMR. This is a weakness as delirium may not have been documented correctly or not at all in the EMR even when present. Third, 4AT screening by the research staff was limited to only three consecutive days in the hospital for each participant, and there was no screening by research staff on the weekends, implying that not all patients have three (consecutive) screening scores. Fourth, according to the study design, we acknowledge that the severity of CI may be different in the two groups, as the two groups were not randomized. Thus, we do not know whether the decrease in use of antipsychotic medications, hypnotics or sedatives are caused by an improved medication practice or lower needs of these medications. However, number of patients with dementia or CI established prior to admission were similar in the two groups and the data collection was performed at the same departments, at the same time of year, and no administrative changes according to admittance rules for these departments were done between the time periods.

Our study also has some strengths. We used validated and established screening instruments to detect patients at risk, and the measures used in the intervention are based on well-known models and guidelines. Additionally, we used an evidence-based quality improvement model to implement the dementia-friendly hospital program and engaged the wards in the development and implementation of the intervention, which may facilitate the incorporation of the program into the routine in these wards and lead to subsequent improvements beyond the project period.

### Implications for clinical practice

Implementing this program has provided benefits for hospitalised older patients with CI and delirium. Furthermore, health care professionals have gained valuable knowledge about how to implement new tools and measures in a challenging, busy environment. From a clinical perspective, systematic and consistent screening with subsequent assessment of whether the CI detected is delirium or long-term cognitive impairment may be of more importance than the choice of screening tool. However, given the complexity and cost associated with managing patients with delirium, the simple 4AT screening tool may help target resources more appropriately.

Further implementation of this program should focus on communication with risk patients’ informal caregivers and community-based health care services on how to recognize and manage delirium, as early detection and management in the community may prevent further hospital admissions.

## Conclusions

Evaluation of the impact and implementation of the dementia-friendly hospital program shows that it is possible to introduce early screening of CI, and significantly improve relevant measures in the management of patients identified with CI and at risk of delirium. This includes increasing the number of non-pharmacological measures and reducing the prescription of antipsychotic, hypnotic and sedative medications. However, the program did not show any significant effect on the detection of patients with CI or delirium.

## Data Availability

The dataset generated and/or analysed during the current study is available from the corresponding author by reasonable request.

## Abbreviations

HELP: Hospital Elder Life Program
ACE: Acute Care for Elders
NICE: National Institute for Health and Care Excellence Guidelines
CI: Cognitive Impairment
PDSA: Plan-do-study-act circle
QI: Quality Improvement
4AT: 4 ‘A’s Test
CAM: Confusion Assessment Measure
EMR: Electronic Medical Records
